# Researches among the Protozoic and Carboniferous Rocks of Central Kentucky, Made during the Summer of 1846

**Published:** 1847-02

**Authors:** 


Art. VII.—*Researches among the Protozoic and Carboniferous Rocks of Central Kentucky, made during the summer of* 1846. By D. D. Owen, M.D., and J. G. Norwood, M.D. St. Louis. 1847.

With a view to clear up some doubtful points in Western Geology the authors of this pamphlet made an excursion, during the last summer, from Nashville, through the central counties of Kentucky, to this city. Leaving Nashville, which stands on the same formation which immediately underlies Lexington and Cincinnati, they passed through Gallatin, in Tennessee, and thence through Scottsville, Glasgow, New Haven, and Bardstown, in this State. The surface rock at Nashville is continued on to Gallatin, and, no doubt, into the fine region about and above Hartsville. This is the Blue limestone, the basis of our fossiliferous rocks, and belongs to the "*Silurian*" system—a term derived from "Silures," the principal tribe of Celts or ancient Britons, who occupied part of Wales and the bordering counties of England, where these ancient fossiliferous strata are most distinctly exhibited. (Lyell.) Whereever this rock comes to the surface the soil appears to be of a superior quality. It has two axes of elevation, one of which is at Nashville, and the other at Cincinnati. It is in this rock, as we have mentioned in another article, that the saline springs of Kentucky are found. At Nashville there is a spring impregnated with the same substances.

The Grey or Cliff limestone, which is so conspicuous on the Falls of the Ohio, and which increases greatly in thickness towards the north, in Tennessee is a very inconsiderable formation. It thins out to a few inches a short distance beyond Nashville, and is not met with in coming from Gallatin towards Scotsville until you approach the confines of Kentucky. This rock, at the Falls, in the opinion of Mr. Verneuil, is divided between the system spoken of, the Silurian, and a newer system, the *Devonian*, so called from a county in England—the lower strata belonging to the former, and the upper, probably from the one containing the Catenipora, to the latter. This system embraces also the Black slate, which is seen soon after you begin to ascend the ridge fifteen miles north of Gallatin.

Above the Black slate the "Encrinital" limestone of Dr. Troost is found, in the same position which it occupies at the Button-mould Knob near Louisville. With it, it will probably be determined, the "Carboniferous" system commences —so called for the reason that it is always above these rocks that coal is found. The carboniferous, or as it is sometimes called, the Mountain limestone, is spread over the Barrens of Kentucky. It is in this formation, and in the upper layers of it, that the Mammoth Cave has been wrought out. The Grayson Springs, as we have said, occur in this formation.

It may be a matter of inquiry with some readers, how the geologist is enabled to determine the relative ages of rocks, and assign them to their proper systems? The organic remains found imbeded in the strata furnish the clue. In the Silurian rocks of England a certain group of fossils have been found; these fossils do not extend into the strata above; the animals appear to have become extinct with the sedimentary deposit which formed the rocks of the Silurian system. When the geologist in Russia or America meets with fossils of the same group—meets, as he often does with fossils of the same identical species—he recognizes the strata as belonging to the same system. We shall not soon forget the pleasure evinced by Mr. Verneuil when we showed him the *Phillipsia*, which we had found in the "Encrinital limestone," and which at once established in his mind the rock as belonging to the Carboniferous system. Our friend, Dr. Shumard, traveled from Louisville to Nashville, last summer, over the same region explored by Drs. Owen and Norwood, a short time before these gentlemen set out on their expedition; and by his knowledge of paleontology he was able to define the position of the various strata. We have now in our possession his letters to us describing the succession of systems as he advanced towards Nashville, just as they are exhibited in the satisfactory and interesting section of Drs. Owen and Norwood.

This ardent young geologist has ascertained that fifty of our Louisville fossils are identical with European species, principally Russian; and that one, the Calymene Blumenbachii, is found in Tennessee, Kentucy, Ohio, New York, in England, Belgium, Germany, Russia, and in Asia and Africa.

Fossils have been beautifully styled "medals of creation." They speak to the paleontologist an intelligible and impressive language. He reads in them the comparative antiquity of rocks and mountains. They disclose to him the first vestiges of animate creation, and the point at which races of beings ceased to exist, and new races came into being. But they also communicate to him practical knowledge. Coal, as we have remarked, is never found under the mountain limestone—the pentremital layer; the geologist knows that it is useless to search for it below the *Pentremite* and the *Archimedes.*

Let us not be charged, in these geological notices, with wandering from our profession; geology has important relations to medicine. Has it never come into the mind of the reader, how closely connected certain causes of disease are with the soil? One day, last spring, we were fossilizing on the upper beds of our Cliff limestone just where it disappears under the Black slate, and stopping at the spring of an intelligent farmer whose land extended to both formations, it occurred to us to ask him on which side was there most sickness among his neighbors. His reply was, as we were prepared to hear it, that they were much more sickly to the west. This was in the region of the black slate. The "Pond Settlement" below Louisville, the sickly character of which is well known, is upon the same rock. The black slate appears to be the favorite abode of fever and ague.

But another fact bearing upon the relations of geology to medicine has occurred to us, in casting our eye over the formations in the valley of the Ohio, and that is, that epidemic cholera, in its sweep over us thirteen years ago, was most fatal in those regions where the Blue limestone is the surface rock. We would cite Lexington, for example, in comparison with Louisville, and the small towns in that quarter of the State as compared with the villages in the Green river country. From Cincinnati to Harrodsburg it is blue limestone, and the malignity of the disease in all that region will long be remembered. At Nashville the same rock reappears, and in some towns in that part of Tennessee the mortality was as great as in any place where the epidemic prevailed. We do not know that there is any value in these facts. The coincidence may have been accidental, but it strikes us as, at least, curious, and worth adverting to. Certain it is that our chances for obtaining a knowledge of the causes of disease will be increased by some attention to geology.

We have read the "Researches" with great pleasure, and hope its authors will be encouraged to pursue their interesting labors, by which they are acquiring reputation, and science many instructive facts.

